# Hemolytic uremic syndrome.

**DOI:** 10.3201/eid0104.950411

**Published:** 1995

**Authors:** M Beers, S Cameron


### Hemolytic Uremic Syndrome

Along with a report of the first outbreak of hemolytic uremic syndrome (HUS) caused by Shiga-like toxin (SLT) producing *E. coli* in Australia (1), this issue of *Emerging Infectious Diseases* presents three papers detailing the investigations of pediatric HUS cases linked to Shiga toxin (ST) and SLT producing bacteria. Goldwater and Bettelheim present a case of pediatric HUS associated with SLT producing *Escherichia coli* (SLTEC) O48:H21 in South Australia; this strain has not previously been recognized as an SLTEC. Saeed et al. report on the increasingly common identification of HUS in Saudi Arabia, its association with multiple-antibiotic-resistant *Shigella dysenteriae* type 1, and the inherent dangers of treating such patients with ampicillin and nalidixic acid. Al-Qawari et al. report on the results of active surveillance for dysentery and HUS in Saudi Arabia and discuss a possibly elevated risk for HUS in patients with bloody diarrhea who are hospitalized and treated with nalidixic acid during an outbreak of *S. dysenteriae* type 1.

The three papers raise a number of important issues regarding HUS. First, it is clear that a large number of SLT producing bacteria have the potential to cause HUS, particularly among children. Current research has focused on *E. coli* O157:H7, which since the early 1980s has emerged as a major foodborne cause of bloody diarrhea, hemorrhagic colitis, and HUS since the early 1980s (5-7). However, the large outbreak of pediatric HUS in South Australia in 1995 caused by foodborne *E. coli* O111:HNM has demonstrated that minor pathogens can emerge as major causes of HUS (1). Goldwater and Bettelheim (5) and other researchers have identified a number of *E. coli* serotypes isolated from patients with HUS (6,7). A-Qawari et al. and Saeed et al. offer a timely reminder that *S. dysenteriae* type 1, a pathogen with a human-only reservoir, is an equally serious contender in HUS etiology and pathogenesis when conditions facilitate the person-to-person transmission of pathogens.

The second key issue raised by the latter two papers concerns treatment of bloody diarrhea. Both discuss the potential for antibiotic (ampicillin and nalidixic acid)-mediated HUS and conclude that this issue should be carefully evaluated before antibiotics are used to manage bloody diarrhea. Saeed et al. note that the wide variety of antibiotics used to treat bloody diarrhea in Saudi Arabia could be explained by the various prescription practices of doctors recruited from different parts of the world. Antibiotic resistance is a worrying component of the mechanisms of emerging infectious diseases. Inappropriate antibiotic use is a key factor in the development of resistance, and major efforts must be directed towards educating physicians on effective prescribing practices.

Central to all three papers is the need for surveillance of organisms that cause bloody diarrhea, hemorrhagic colitis, and HUS, as well as for knowledge of the local epidemiology of SLTECs, their potential sources, and the optimal way to investigate and manage outbreaks. Goldwater and Bettelheim discuss the characteristic disappearance of *E. coli* from patients' stools after the development of HUS and, therefore, the importance of early detection in cases of bloody diarrhea. Laboratory testing of bloody diarrheal specimens is clearly critical to understanding the epidemiology of toxin-producing organisms that relate to the development of HUS (8). However, testing can be difficult, time-consuming and costly. Not all laboratories routinely test for SLTECs or have the capacity to do so. In Australia, for example, using polymerase chain reaction (PCR) technology to detect SLT genes is expensive: a negative test result costs approximately $A15, but if the results are positive, the cost rises to around $A250 when the SLTEC is isolated and typed.

Human surveillance is essential to the early detection of outbreaks and to the critical assessment of the impact on public health of new approaches to food safety (2,8). We conservatively estimate that the South Australian HUS outbreak has cost around $A20 million in direct and indirect costs, with major impacts being felt by industry. This must surely be considered when contemplating the costs of surveillance. One approach may be to use PCR techniques on all samples of bloody diarrhea in children under the age of 16 because if surveillance is to be effective, it must be specific (9). Intermittent surveys, or the use of sentinel laboratories for all cases of diarrhea (8) could be undertaken and mandatory notification of HUS instituted.

Compulsory notification of *Shigella* infection is a requirement in Australia, and including SLTECs on the list of notifiable diseases is being considered. A national surveillance scheme for HUS was established in 1994, although notification is not mandatory. Without formal notification requirements, good reporting of HUS has been associated with either clustering of cases or the fact that few hospitals in a region have the capacity to manage these cases (10).

Although more attention has been focused on *E. coli* O157:H7, S. *dysenteriae* is likely the most common cause of HUS in children worldwide and more attention needs to be given to this pathogen in terms of surveillance and control. A strong and healthy public health infrastructure is required to address the infectious disease issues raised by HUS (11).

**Mary Beers,* Scott Cameron†**
*National Centre for Epidemiology and Population Health, and South Australian Health Commission; †Communicable Disease Control Unit, South Australian Health Commission, Adelaide, Australia

### References:

1. Cameron AS, Beers MY, Walker CC, et al. Community outbreak of hemolytic uremic syndrome attributable to *Escherichia coli* O111:NM—South Australia, 1995. MMWR 1995;44:550-8.
2. Waters JR, Sharp JC, Dev VJ. Infection caused by *Escherichia coli* O157:H7 in Alberta, Canada, and in Scotland: a five-year review, 1987-1991. Clin Infect Dis 1994;19:834-43.
3. Bell BP, Goldoft M, Griffin P, Davis MA, Gordon DC, Tarr P, et al. A multistate outbreak of *Escherichia coli* O157:H7-associated bloody diarrhea and hemolytic uremic syndrome from hamburgers: the Washington experience. JAMA 1994;272:1349-53.
4. Alexander ER, Boase J, Davis M, et al. *Escherichia coli* O157:H7 outbreak linked to commercially distributed dry-cured salami—Washington and California, 1994. MMWR 1995;44:157-60.
5. Goldwater PN, Bettelheim KA. The role of enterohaemorrhagic *Escherichia coli* serotypes other than O157:H7 as causes of disease in Australia. Communicable Diseases Intelligence 1995;19:2-4.
6. Caprioli A, Luzzi I, Rosmini F, Resti C, Edefonti A, Perfumo F, et al. Communitywide outbreak of hemolytic-uremic syndrome associated with non-O157 verocytotoxin-producing *Escherichia coli*. J Infect Dis 1994;169:208-11.
7. Griffin PM, Tauxe RV. The epidemiology of infections caused by *Escherichia coli* O157:H7, other enterohaemorrhagic *E. coli*, and the associated hemolytic uremic syndrome. Epidemiol Rev 1991;13:60-98.
8. Alexander ER. Editorial response: surveillance of *Escherichia coli* O157:H7—a necessity for the prevention of an emerging infectious disease. Clin Infect Dis 1994;19:844-5.
9. Satcher D. Emerging infections: getting ahead of the curve. Emerging Infectious Diseases 1995;1:1-6.
10. Siegler RL, Pavia AT, Christofferson RD, Milligan MK. A 20-year population-based study of postdiarrheal hemolytic uremic syndrome in Utah. Pediatrics 1994;94:35-40.
11. MacDonald KL, Osterholm MT. The emergence of *Escherichia coli* O157:H7 infection in the United States: the changing epidemiology of foodborne disease. JAMA 1993;269:2264-6.

